# A Rare Presentation of Left Ventricular False Tendon Across the Mitral Valve Connecting the Left Atrium and Ventricle: A Case Report

**DOI:** 10.2174/011573403X395039250725103753

**Published:** 2025-08-05

**Authors:** Fazeela Ansari, Alma AlFakhori, Montaser Nabeeh Al Smady, Mohamed Kasem, Ahmed Adel Hassan

**Affiliations:** 1 College of Medicine, Dubai Medical University, Dubai, United Arab Emirates;; 2 College of Medicine, Mohammed Bin Rashid University of Medicine and Health Sciences (MBRU), Dubai Health, Dubai, United Arab Emirates;; 3 Cardiology Department, Al Jalila Children’s Hospital (AJCH), Dubai Health, Dubai, United Arab Emirates

**Keywords:** Left ventricular false tendon, cardiac false tendon, palpitations, mitral regurgitation, ventricular arrhythmias, transthoracic echocardiography

## Abstract

**Introduction:**

Cardiac False tendons are anatomical variants of fibromuscular structures that generally should not exist in the heart. The left ventricular tendon, which crosses the left ventricular cavity, is typically seen in most cases of false cardiac tendons; however, our case differs from other cardiac false tendons. This case involves a fibrous tendon that spans the mitral valve, connecting the left atrium and ventricle. This unusual presentation has only been documented once. Based on our knowledge and research, this is the first documented occurrence in the United Arab Emirates and the second incident worldwide.

**Case Presentation:**

A 12-year-old girl presented to an outpatient clinic with palpitations as her main complaint. After transthoracic echocardiography, it was suggested that a false tendon was passing through the mitral valve to connect the left atrium and left ventricle. We performed a transesophageal echo to confirm the diagnosis, and the results were identical to those of the transthoracic echocardiography. Since the uncommon false tendon only caused mild symptoms, an extensive multidisciplinary meeting was held, and we opted to manage the patient conservatively and monitor them annually at the outpatient cardiology clinic.

**Conclusion:**

In this uncommon case presentation, accurately diagnosing the condition and establishing a treatment and follow-up plan are crucial for understanding similar cases in the future. This variant of false tendons across the mitral valve appears to be a benign anatomical structural variant, comparable to other false tendons in the heart. Currently, there is insufficient evidence to link false tendons to increased cardiac morbidity and mortality.

## 1 INTRODUCTION

In 1893, Turner initially reported the Left Ventricle False Tendon (LVFT) and observed it during dissection, but the functional significance of these structures remains unresolved, mainly [1]. These false tendons are typically benign anatomical variants of fibromuscular or fibroid endocavitary structures, characterized by variations in origin, papillary muscles, conduction tissue fibers, and communication with the ventricular free wall [2]. Numerous reports of this false tendon in the left ventricle have been made worldwide [3]. However, this case represents a rare finding of an additional variant of a fibrous or fibromuscular band that connects from the interatrial septum of the left atrium to the left ventricle through the mitral valve, a condition previously reported only once [4]. This is the first documented case of this LVFT variant within the United Arab Emirates (UAE), the Middle East region, and the second worldwide.

## 2 CASE PRESENTATION

We present a 12-year-old female referred to our outpatient pediatric cardiology clinic at a tertiary care hospital for evaluation of palpitations. The patient had a history of chronic recurrent abdominal pain, and an upper gastrointestinal endoscopy was done, and biopsies were taken from the lower esophagus, stomach, and duodenum. A stomach biopsy revealed gastric mucosa with moderate chronic active gastritis, and *Helicobacter pylori*-like organisms were identified using a Giemsa stain. The patient was subsequently treated for this condition. The patient was also noted to have a history of heavy menstrual bleeding and was following up with obstetrics and gynecology regarding this.

The patient is a tall girl with a height of 171.5 cm and a thin stature for her age, which raised suspicion of Marfan syndrome. During the clinical assessment, her vitals were within the standard reference range for her age, and the only noteworthy finding was a faint systolic murmur that was not accompanied by thrills or heaves.

Laboratory investigations revealed microcytic hypochromic anemia, with low iron studies and an initial hemoglobin level of 9.3 g/dL, which decreased to 8.7 g/dL at the 6-month follow-up. It is being treated with iron supplementation, but has yet to resolve and is likely correlated with her history of heavy menstrual bleeding. Moreover, all the remaining laboratory results were unremarkable.

Due to the presence of a faint systolic murmur, a transthoracic echocardiogram (TTE) was warranted for further evaluation. The TTE revealed a tendon originating from the interatrial septum, extending posteriorly across the mitral valve, and attaching to the lateral wall of the left ventricle, as suspected. A transesophageal echocardiogram (TEE) was performed to obtain a clearer view and for a more comprehensive assessment. It confirmed the findings and revealed mild mitral valve prolapse (MVP) and moderate mitral regurgitation (MR) in two jets. No non-compaction cardiomyopathy was identified during TTE or TEE; as such, the patient was not subjected to a Magnetic Resonance Imaging (MRI). Figs. (**1**-**4**) depict the images obtained during the TEE, and Table **1** describes the echocardiographic measurements. Furthermore, Video 1 presents a Doppler 4-chamber view of TEE with the LVFT (Supplementary material).

Furthermore, to assess the complaints of palpitations, a 24-hour Holter study was warranted and done. It showed sinus tachycardia with a maximum heart rate reaching 200 beats per minute (bpm) during activity, and a daily average heart rate of 115 bpm. While asleep, the average heart rate was 94 bpm, without any supraventricular tachycardia (SVTs) or ventricular tachycardia (VTs). Two Premature Ventricular Contractions (PVCs) and 8 Premature Atrial Contractions (PACs) were recorded, which accounted for <0.1%, deeming them insignificant.

A follow-up Holter study was performed after 6 months, which showed an average heart rate of 94 bpm during the day, 84 bpm during the night, and 91 bpm over the complete recording. The maximum heart rate was 196 bpm, and the minimum was 49 bpm. The standard deviation over all regular beats was 158.5 Normal sinus rhythm was observed in the recordings without any Supraventricular extrasystole (SVES), Ventricular Ectopics (VES), SVT, and VT. Sinus tachycardias between 150-167 bpm were observed in the recordings in the evening within a 1-hour period. However, since the average heart rate during the day was 94, it was observed that it did not meet sufficient criteria to be classified as inappropriate sinus tachycardia. Furthermore, the patient had no complaints about tachycardia or palpitations.

Due to an association between connective tissue disorders and MR, and based on the suspected Marfan habitus and MR in our patient, single gene sequencing of FBN1 was performed, which was reported to be negative as no clinically relevant sequence variants were identified. Furthermore, the general pediatrician ruled out other connective tissue disorders during their assessment, supported by negative serum antibodies, including ANA, and the geneticist did not suggest any other possible connective tissue disorders.

The initial management plan was to monitor this patient conservatively in the outpatient department with a Holter monitor, a cardiac echo annually, and a TEE every five years. During her most recent follow-up visit, propranolol 20 mg was prescribed twice daily, as needed, to help manage the patient's palpitations and tachycardia.

## 3 DISCUSSION

Embryologically, LVFTs are thought to originate from the non-compact inner myocardial layer of the developing heart. The inner layer comprises trabeculations forming irregular ridges within the ventricular cavity, which are believed to be the origin of false tendons [2]. Although the prevalence of LVFTs in the general population has not been documented, various studies have reported a wide range of prevalence, from 0.4% to 83% [5, 6]. A recent morphological-based echocardiography study identified that LVFTs were more common in males than females [3].

Transthoracic and transesophageal echocardiograms are quite effective in diagnosing false tendons. In our patient, when assessing with both TTE and TEE, we discovered the false tendon crossing the mitral valve. The significance of a false tendon crossing the mitral valve is unclear, as this is an uncommon finding, and the data from the literature poorly describe this specific variant. The false tendon across the mitral valve causes minimal symptoms, with mitral valve prolapse (MVP) and mitral regurgitation (MR) [7].

An association between connective tissue diseases, MVP, and MR exists, where the mitral valve tends to be damaged or malformed, leading to mitral valve dysfunction. Several connective tissue disorders have been implicated as causes of MVP and MR, such as Marfan syndrome, Ehlers-Danlos syndrome (EDS), systemic lupus erythematosus (SLE), Larsen-like syndrome, and Loeys-Dietz syndrome (LDS) [8, 9]. It is imperative to consider connective tissue disorders as a cause, especially when physical manifestations, such as our patient's body habitus, are present.

Although the exact mechanism underlying false tendon-induced palpitations is undetermined, potential pathways include active stretching of the Purkinje fiber network on the interventricular septum or conduction through the false tendon [4, 8]. Left ventricular false tendons (LVFTs) have been previously correlated with the origin of premature ventricular contractions (PVCs) [8], which our patient had in her initial Holter study. Nevertheless, the risk of progression to malignant PVCs is deemed insignificant [3]. However, it is imperative to recognize that LVFTs are associated with early repolarization, and the risk of ventricular arrhythmias exists, which is crucial when considering management options and counseling patients and their families about the possibility of ventricular arrhythmias. Evidence supporting that the palpitations experienced by our patient are secondary to this false tendon is highly likely. Still, it cannot be stated as the isolated cause due to the overlapping and potentially propagating factor of her unresolved anemia.

Although ablation is typically used to control and treat false left ventricular tendons [10], the management of this specific variant of LVFT is still unknown [11]. After a multidisciplinary team discussion, the cardiology team decided to monitor this patient conservatively in the outpatient clinic, prescribing propranolol 20 mg as needed to help manage palpitations and tachycardia. However, alternative therapeutic strategies will be considered if any new or worsening symptoms arise or if the mitral regurgitation deteriorates [12].

## CONCLUSION

Left ventricular false tendons (LVFTs) are commonly observed, especially in young, non-obese individuals. This particular variant, spanning the mitral valve, is generally considered a benign anatomical variant, much like other false tendons found within the heart. Although a likely association exists between these false tendons, palpitations, and mitral regurgitation (MR), definitive proof remains lacking. While current evidence does not support a direct link between false tendons and increased cardiac morbidity or mortality, it remains important to remain vigilant regarding their potential role in ventricular arrhythmias.

## AUTHORS’ CONTRIBUTIONS

The author confirms their contribution to the paper as follows: draft manuscript: AAF, FA and MNAS; study concept or design: AAH and MK. All authors reviewed the article and approved the final version of the article.

## Figures and Tables

**Fig. (1):**
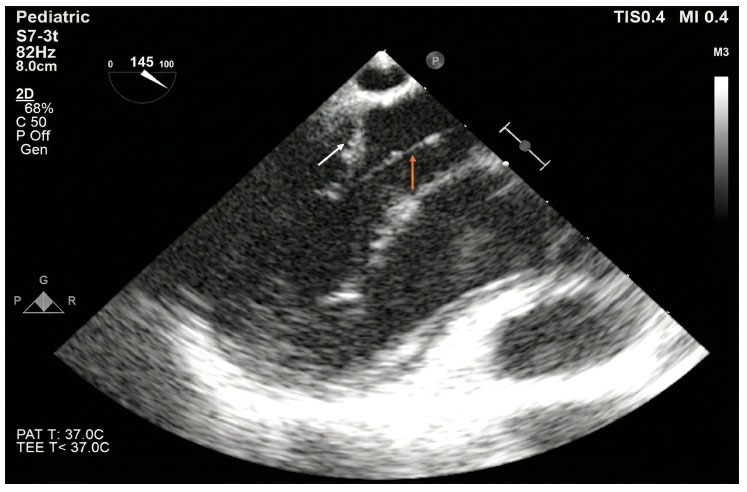
A transesophageal echo at 145 degrees showing the false tendon (orange arrow) during diastole extending from the left atrium to the left ventricle. Mitral valve (white arrow).

**Fig. (2):**
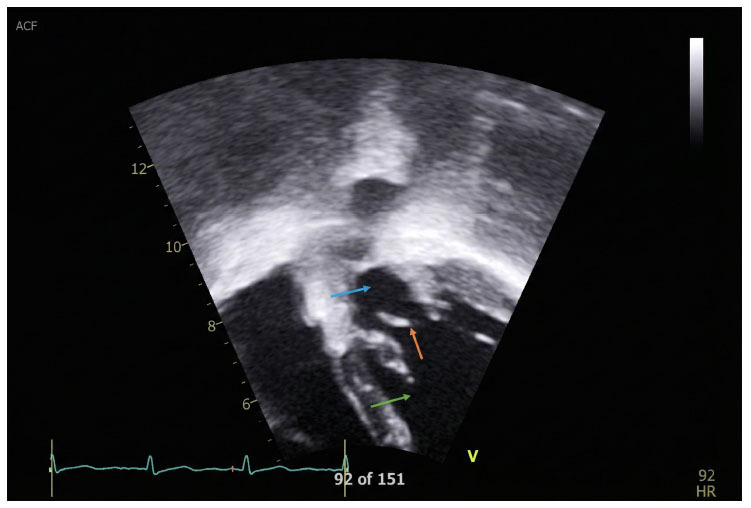
A transesophageal 2D echo with apical 4-chamber view showing the false tendon (orange arrow) during diastole traversing the mitral valve from left atrium (blue arrow) to left ventricle (green arrow).

**Fig. (3):**
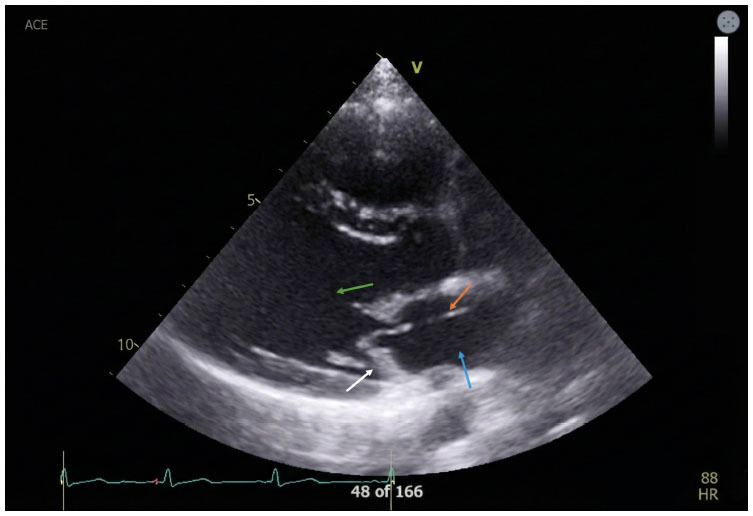
A transesophageal 2D echo with long-axis parasternal view showing the false tendon (orange arrow) traversing the mitral valve (white arrow) from the left atrium (blue arrow) to the left ventricle (green arrow).

**Fig. (4):**
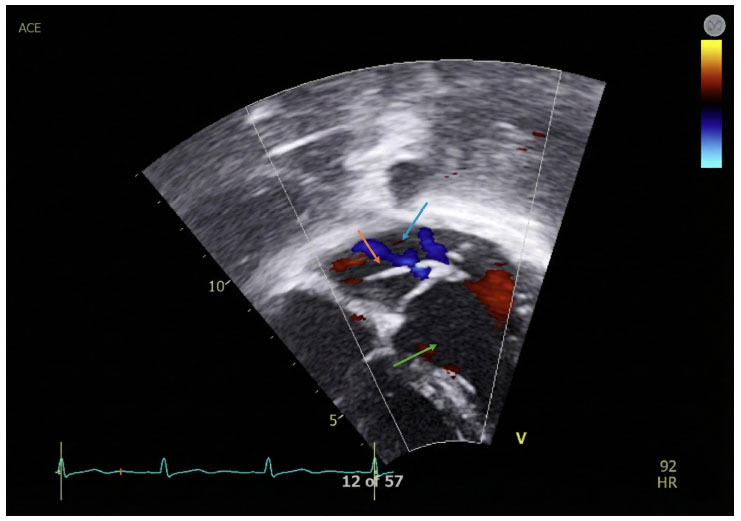
A transesophageal 2D echo with doppler with apical four-chamber view showing false tendon (orange arrow) during systole traversing the mitral valve from left atrium (blue arrow) to left ventricle (green arrow).

**Table 1:** Echocardiographic measurements.

**Parameter (Abbreviation)**	**Value**
Interventricular Septum in Diastole (IVSd)	0.5 cm
Interventricular Septum in Systole (IVSs)	0.6 cm
Left Ventricular Internal Diameter in Diastole (LVIDd)	4.6 cm
Left Ventricular Internal Diameter in Systole (LVIDs)	2.9 cm
Left Ventricular Posterior Wall in Diastole (LVPWd)	0.5 cm
Left Ventricular Posterior Wall in Systole (LVPWs)	0.7 cm
End-Diastolic Volume - Teichholz Method (EDV [Teich])	99 ml
End-Systolic Volume - Teichholz Method (ESV [Teich])	33 ml
Stroke Volume - Teichholz Method (SV [Teich])	65 ml
Ejection Fraction - Teichholz Method (EF [Teich])	66%
Fractional Shortening (%FS)	36%
Relative Wall Thickness (RWT)	0.22

## Data Availability

All data generated or analyzed during this study are included in this published article.

## LIST OF ABBREVIATIONS

LVFT: Left Ventricle False Tendon
MR: Mitral Regurgitation
MVP: Mitral Valve Prolapse
PACs: Premature Atrial Contractions
SVES: Supraventricular Extrasystole
TEE: Transesophageal Echocardiogram
TTE: Transthoracic Echocardiogram
VES: Ventricular Ectopics

## STANDARDS OF REPORTING

CARE guidelines were followed.
